# Supplementary material for: Synthesis of 4,5-Dihydro-1H-[1,2]dithiolo[3,4-c]quinoline-1-thione Derivatives and Their Application as Protein Kinase Inhibitors
Source: Molecules. 2022 Jun 23;27(13):4033. doi: 10.3390/molecules27134033 (PMC9268448; doi:10.3390/molecules27134033)
Supplement: Supplementary file 1 [file molecules-27-04033-s001.zip › molecules-1763413-supplementary.pdf]

**Data Filename**	0018.d	**Sample Name**	
**Sample Type**	Sample	**Position**	Vial 38
**Instrument Name**	Instrument 1	**User Name**	
**Acq Method**	ACN-H2O_60-40.m	**Acquired Time**	3/12/2022 3:32:43 PM
**IRM Calibration Status**	Success	**DA Method**	111.m
**Comment**			

**Sample Group**		**Info.**	
**Stream Name**	LC 1	**Acquisition SW Version**	6200 series TOF/6500 series Q-TOF B.06.01 (B6172 SP1)

User Chromatograms

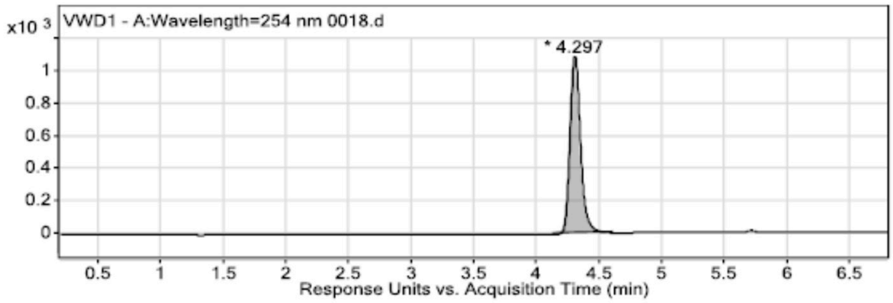

Integration Peak List

Peak	Start	RT	End	Height	Area	Area %
1	4.13	4.297	4.61	1087.31	6233.36	100

User Spectra

Fragmentor Voltage	Collision Energy	Ionization Mode
191	0	ESI

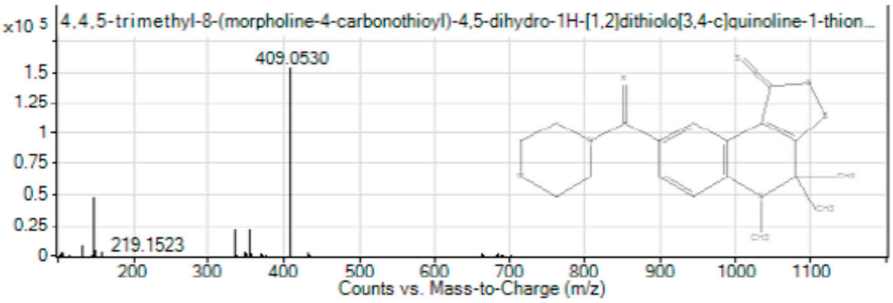

Figure S1: HPLC–HRMS–ESI spectra of 4,4,5-trimethyl-8-(morpholin-4-ylcarbonothioyl)-4,5-dihydro-1H-[1,2]dithiolo[3,4-c]quinoline-1-thione 2a

**Data Filename**	0013.d	**Sample Name**	
**Sample Type**	Sample	**Position**	Vial 33
**Instrument Name**	Instrument 1	**User Name**	
**Acq Method**	ACN-H2O_60-40.m	**Acquired Time**	3/12/2022 2:33:50 PM
**IRM Calibration Status**	Success	**DA Method**	111.m
**Comment**			

**Sample Group**		**Info.**	
**Stream Name**	LC 1	**Acquisition SW Version**	6200 series TOF/6500 series Q-TOF B.06.01 (B6172 SP1)

User Chromatograms

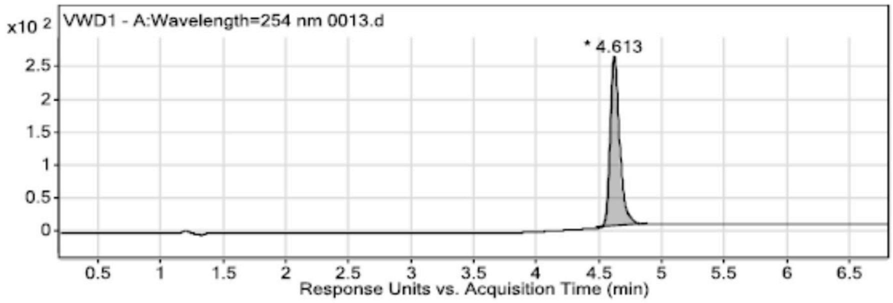

Integration Peak List

Peak	Start	RT	End	Height	Area	Area %
1	4.473	4.613	4.887	258.96	1372.07	100

User Spectra

Fragmentor Voltage	Collision Energy	Ionization Mode
191	0	ESI

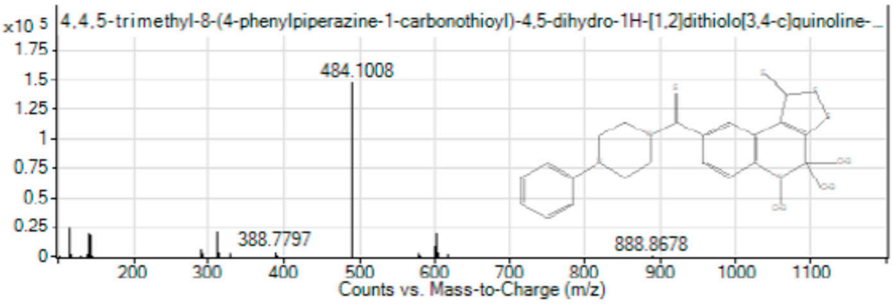

Figure S2: HPLC–HRMS–ESI spectra of 4,4,5-trimethyl-8-[(4-phenylpiperazin-1-yl)carbonothioyl]-4,5-dihydro-1H-[1,2]dithiolo[3,4-c]quinoline-1-thione 2b

**Data Filename**	0004.d	**Sample Name**	
**Sample Type**	Sample	**Position**	Vial 24
**Instrument Name**	Instrument 1	**User Name**	
**Acq Method**	ACN-H2O_60-40.m	**Acquired Time**	3/12/2022 12:41:37 PM
**IRM Calibration Status**	Success	**DA Method**	111.m
**Comment**			

**Sample Group**		**Info.**	
**Stream Name**	LC 1	**Acquisition SW Version**	6200 series TOF/6500 series Q-TOF B.06.01 (B6172 SP1)

User Chromatograms

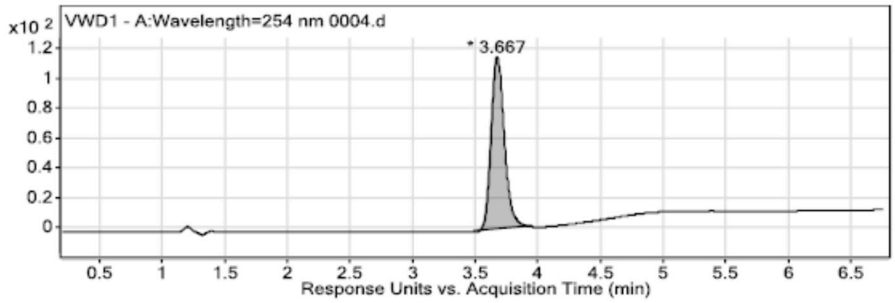

Integration Peak List

Peak	Start	RT	End	Height	Area	Area %
1	3.483	3.667	3.95	115.83	858.22	100

User Spectra

**Fragmentor Voltage**	**Collision Energy**	**Ionization Mode**
191	0	ESI

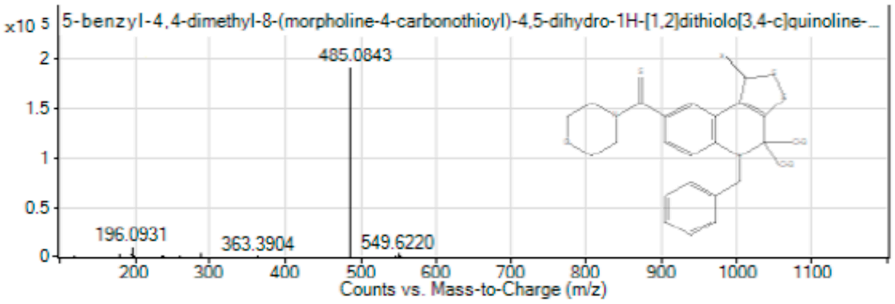

Figure S3: HPLC–HRMS–ESI spectra of 5-benzyl-4,4-dimethyl-8-(morpholin-4-ylcarbonothioyl)-4,5-dihydro-1H-[1,2]dithiolo[3,4-c]quinoline-1-thione 2c,

**Data Filename**	0010.d	**Sample Name**	
**Sample Type**	Sample	**Position**	Vial 30
**Instrument Name**	Instrument 1	**User Name**	
**Acq Method**	ACN-H2O_60-40.m	**Acquired Time**	3/12/2022 1:58:09 PM
**IRM Calibration Status**	Success	**DA Method**	111.m
**Comment**			

**Sample Group**		**Info.**	
**Stream Name**	LC 1	**Acquisition SW Version**	6200 series TOF/6500 series Q-TOF B.06.01 (B6172 SP1)

User Chromatograms

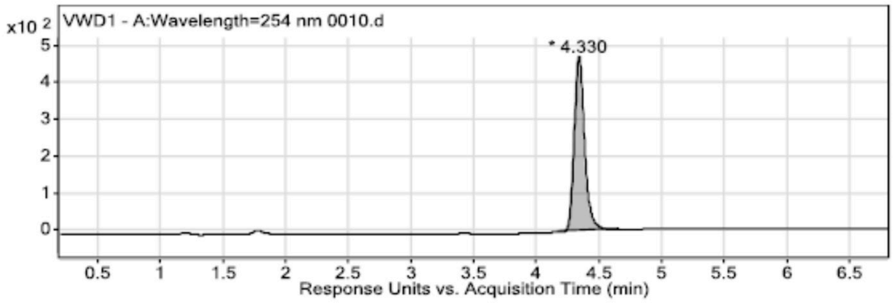

Integration Peak List

Peak	Start	RT	End	Height	Area	Area %
1	4.167	4.33	4.66	472.6	2709.3	100

User Spectra

Fragmentor Voltage	Collision Energy	Ionization Mode
191	0	ESI

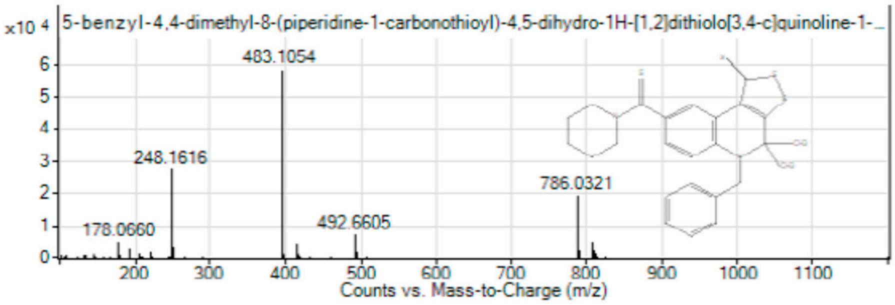

Figure S4: HPLC–HRMS–ESI spectra of 5-benzyl-4,4-dimethyl-8-(piperidin-1-ylcarbonothioyl)-4,5-dihydro-1H-[1,2]dithiolo[3,4-c]quinoline-1-thione 2d.,

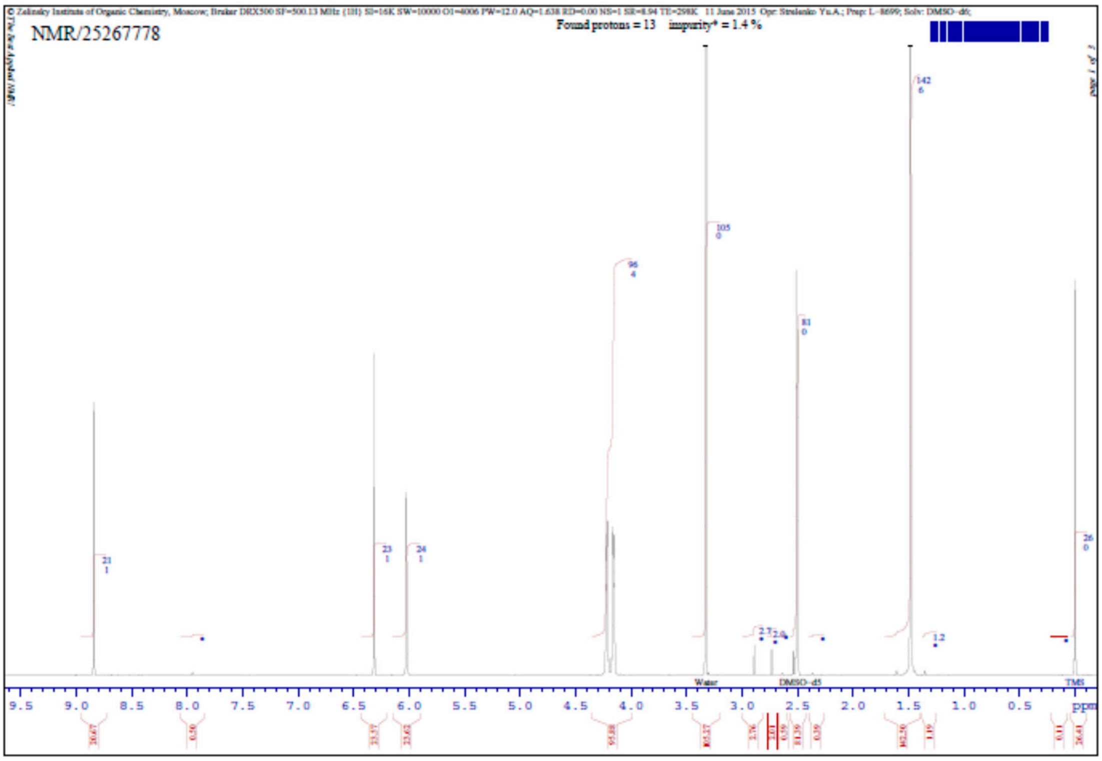

Figure S5: NMR 1H spectra of 4,4-dimethyl-4,5,8,9-tetrahydro-1H-[1,4]dioxino[2,3-g][1,2]dithiolo[3,4-c]quinoline-1-thione 2h,

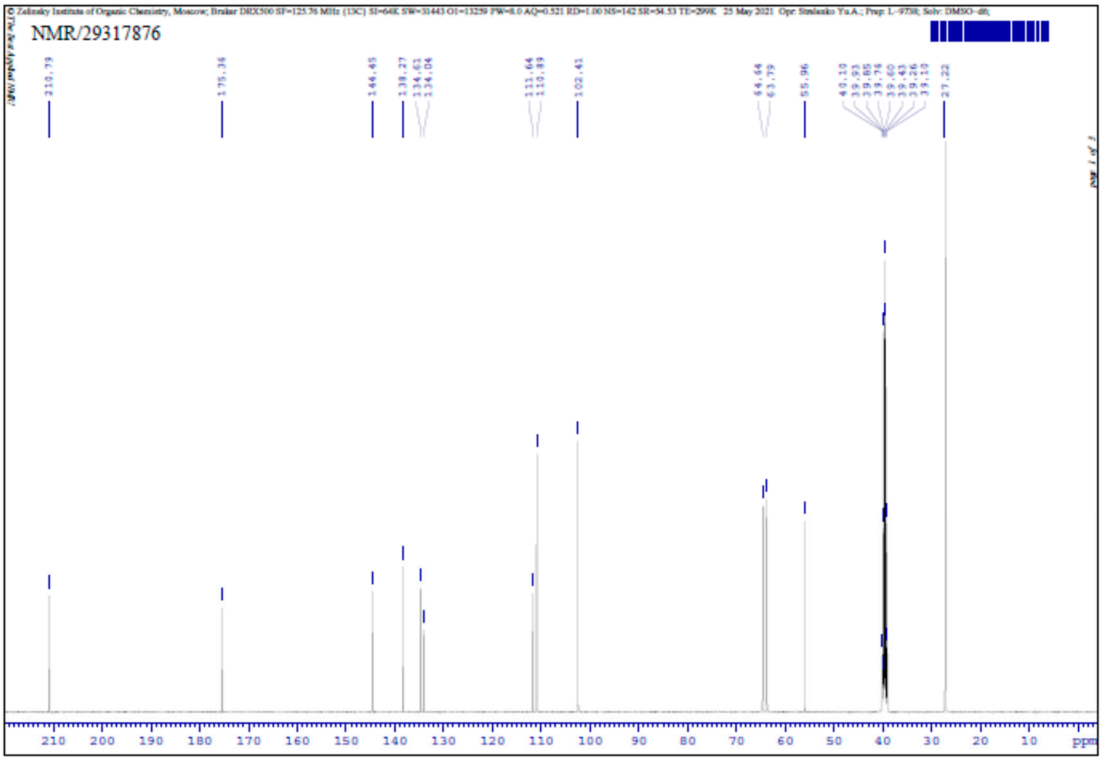

Figure S6: NMR 13C spectra of 4,4-dimethyl-4,5,8,9-tetrahydro-1H-[1,4]dioxino[2,3-g][1,2]dithiolo[3,4-c]quinoline-1-thione 2h,

**Data Filename**	0042.d	**Sample Name**	
**Sample Type**	Sample	**Position**	Vial 68
**Instrument Name**	Instrument 1	**User Name**	
**Acq Method**	ACN-H2O_60-40.m	**Acquired Time**	3/14/2022 2:00:55 PM
**IRM Calibration Status**	Success	**DA Method**	111.m
**Comment**			

**Sample Group**		**Info.**	
**Stream Name**	LC 1	**Acquisition SW Version**	6200 series TOF/6500 series Q-TOF B.06.01 (B6172 SP1)

User Chromatograms

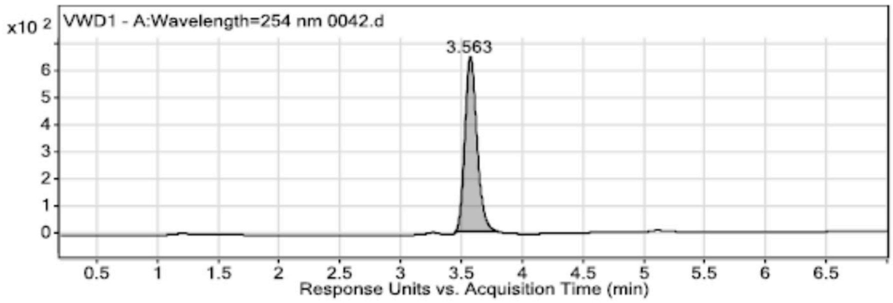

Integration Peak List

Peak	Start	RT	End	Height	Area	Area %
1	3.442	3.563	3.8	651.31	4529.68	100

User Spectra

Fragmentor Voltage	Collision Energy	Ionization Mode
191	0	ESI

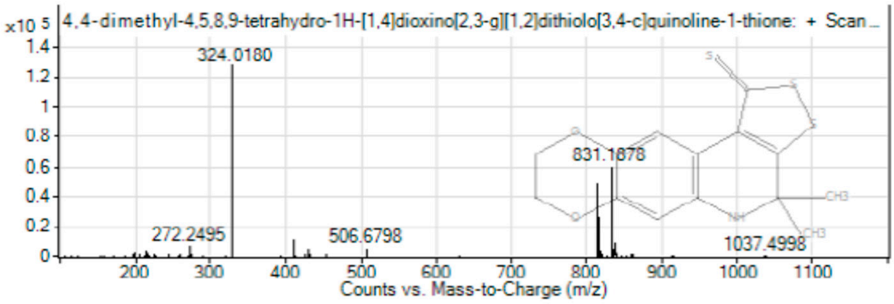

Figure S7: HPLC–HRMS–ESI spectra of 4,4-dimethyl-4,5,8,9-tetrahydro-1H-[1,4]dioxino[2,3-g][1,2]dithiolo[3,4-c]quinoline-1-thione 2h,

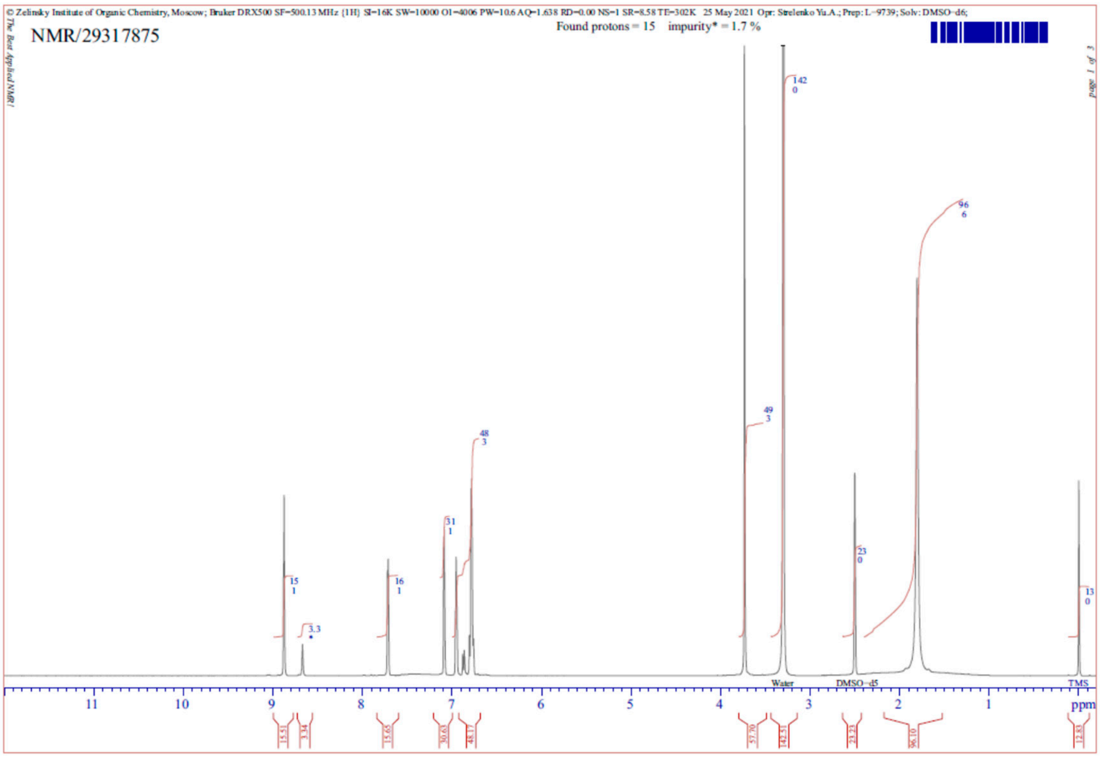

Figure S8: NMR 1H spectra of 8-methoxy-4,4-dimethyl-5-(2-thienylcarbonyl)-4,5-dihydro-1H-[1,2]dithiolo[3,4-c]quinoline-1-thione 2i,

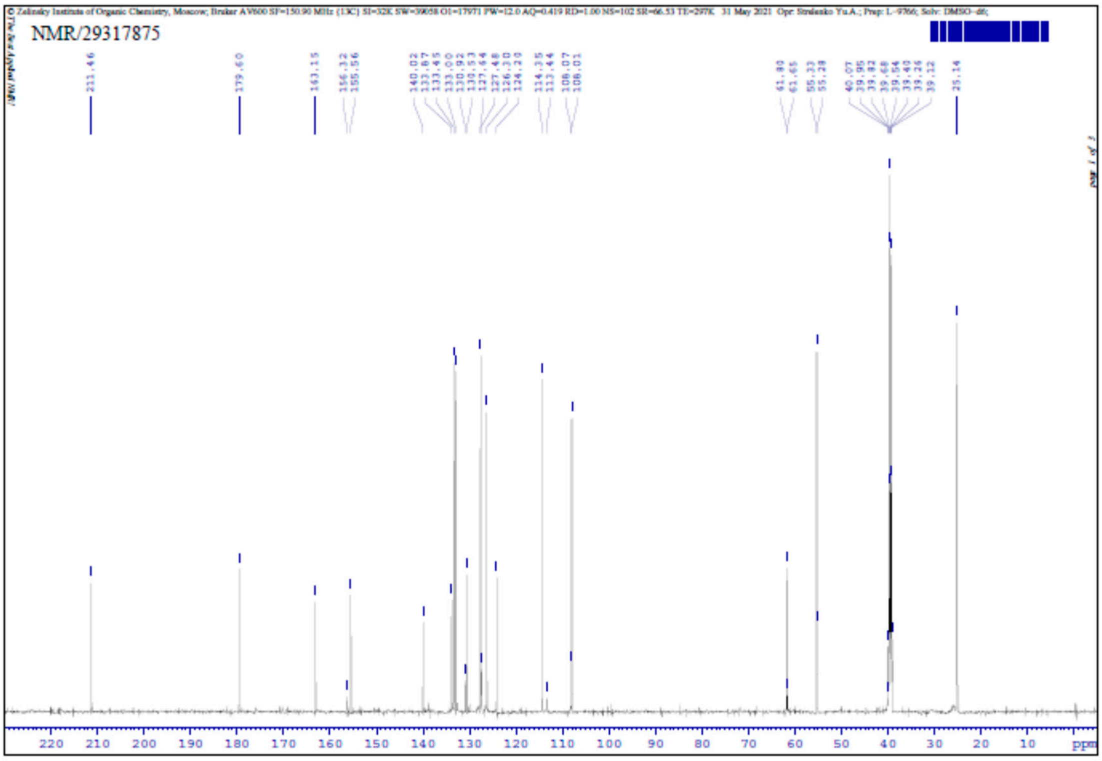

Figure S9: NMR 13C spectra of 8-methoxy-4,4-dimethyl-5-(2-thienylcarbonyl)-4,5-dihydro-1H-[1,2]dithiolo[3,4-c]quinoline-1-thione 2i,

**Data Filename**	0033.d	**Sample Name**	
**Sample Type**	Sample	**Position**	Vial 59
**Instrument Name**	Instrument 1	**User Name**	
**Acq Method**	ACN-H2O_60-40.m	**Acquired Time**	3/14/2022 12:13:01 PM
**IRM Calibration Status**	Success	**DA Method**	111.m
**Comment**			

**Sample Group**		**Info.**	
**Stream Name**	LC 1	**Acquisition SW Version**	6200 series TOF/6500 series Q-TOF B.06.01 (B6172 SP1)

User Chromatograms

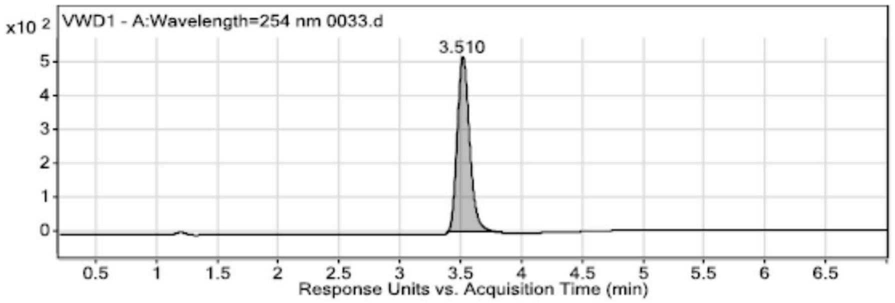

Integration Peak List

Peak	Start	RT	End	Height	Area	Area %
1	3.382	3.51	3.842	518.95	3677.09	100

User Spectra

Fragmentor Voltage	Collision Energy	Ionization Mode
191	0	ESI

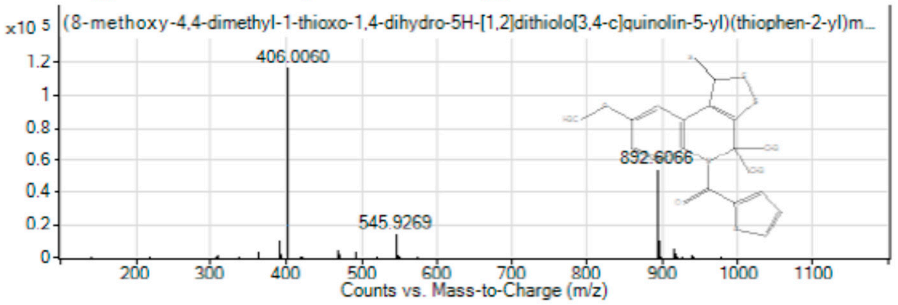

Figure S10: HPLC–HRMS–ESI spectra of 8-methoxy-4,4-dimethyl-5-(2-thienylcarbonyl)-4,5-dihydro-1H-[1,2]dithiolo[3,4-c]quinoline-1-thione 2i,

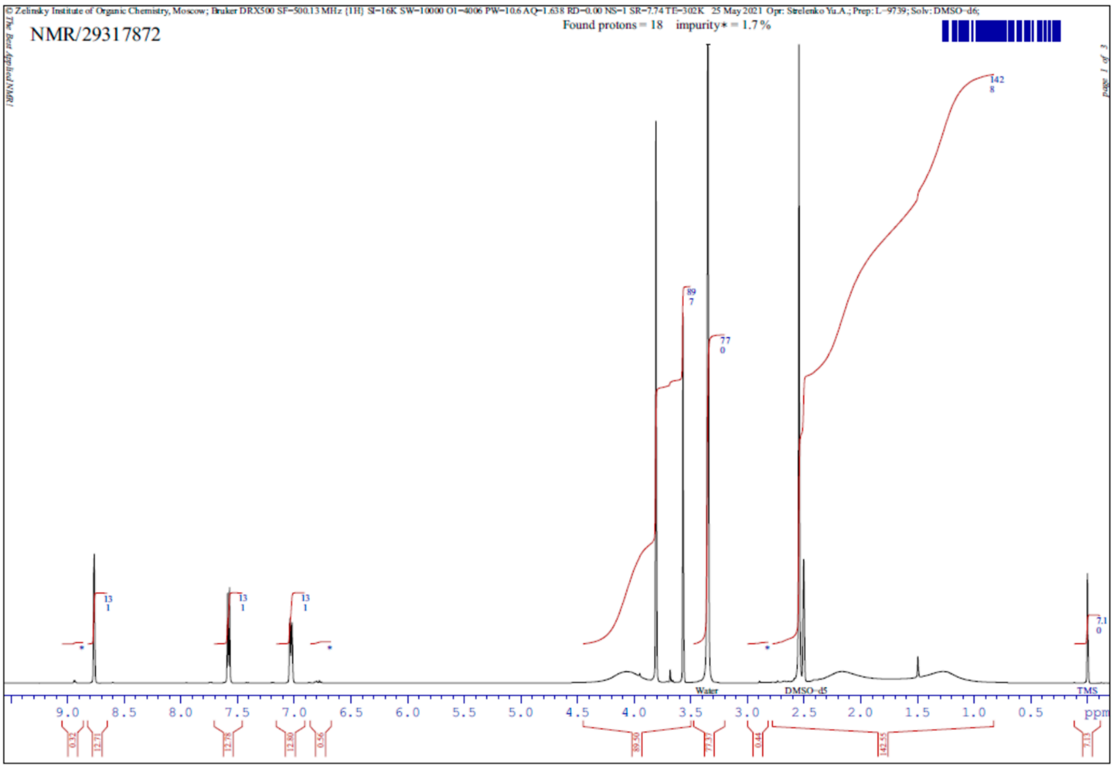

Figure S11: NMR 1H spectra 1-[2-(8-methoxy-4,4-dimethyl-1-thioxo-1,4-dihydro-5H-[1,2]dithiolo[3,4-c]quinolin-5-yl)-2-oxoethyl]pyrrolidine-2,5-dione 2j,

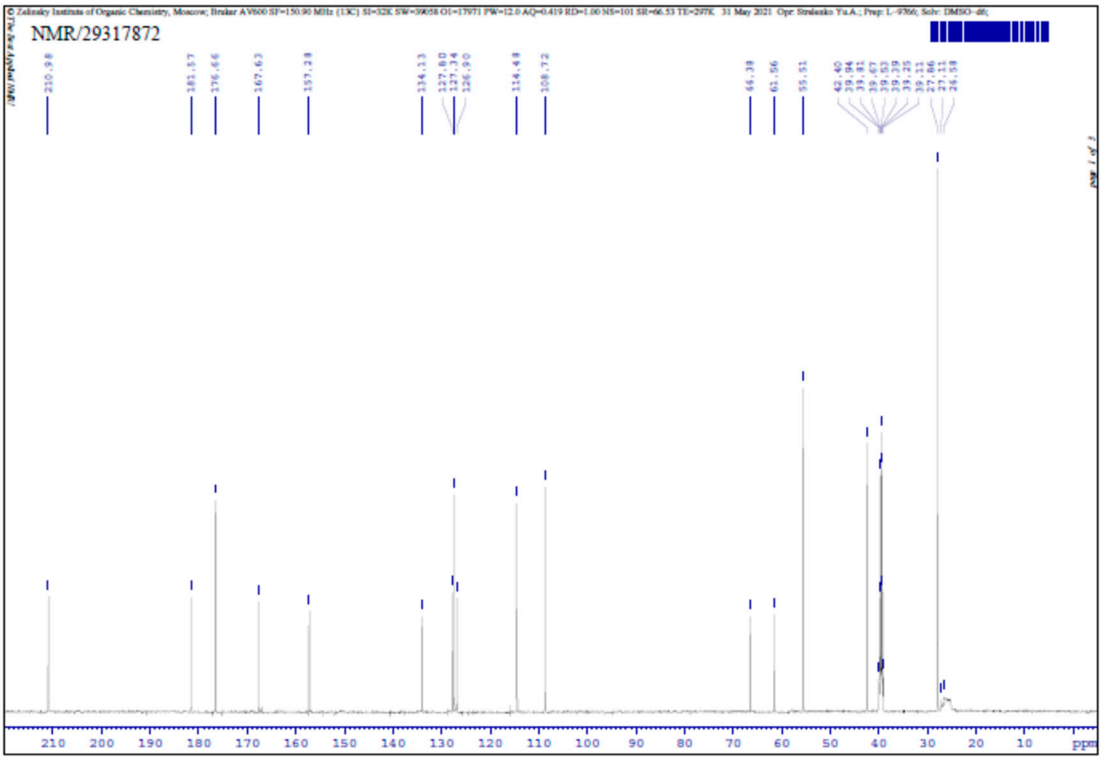

Figure S12: NMR 13C spectra of 1-[2-(8-methoxy-4,4-dimethyl-1-thioxo-1,4-dihydro-5H-[1,2]dithiolo[3,4-c]quinolin-5-yl)-2-oxoethyl]pyrrolidine-2,5-dione 2j,

**Data Filename**	0028.d	**Sample Name**	
**Sample Type**	Sample	**Position**	Vial 54
**Instrument Name**	Instrument 1	**User Name**	
**Acq Method**	ACN-H2O_60-40.m	**Acquired Time**	3/14/2022 11:13:07 AM
**IRM Calibration Status**	Success	**DA Method**	111.m
**Comment**			

**Sample Group**		**Info.**	
**Stream Name**	LC 1	**Acquisition SW Version**	6200 series TOF/6500 series Q-TOF B.06.01 (B6172 SP1)

User Chromatograms

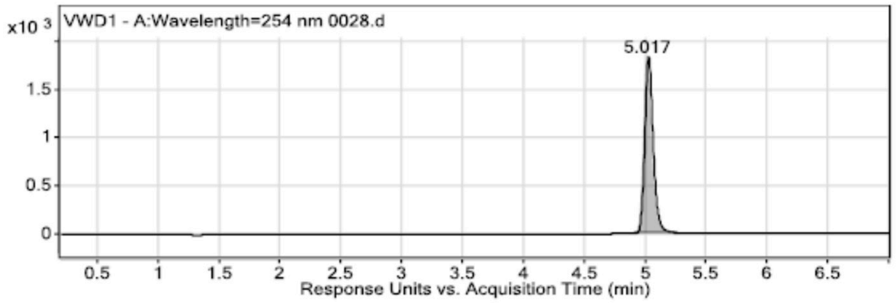

Integration Peak List

Peak	Start	RT	End	Height	Area	Area %
1	4.913	5.017	5.26	1837.64	8894.43	100

User Spectra

Fragmentor Voltage	Collision Energy	Ionization Mode
191	0	ESI

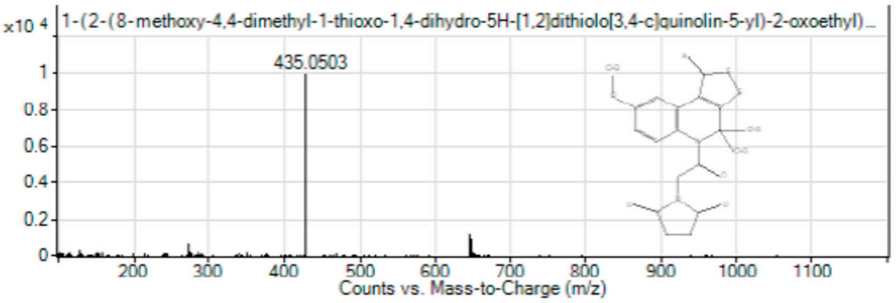

Figure S13: HPLC–HRMS–ESI spectra of 1-[2-(8-methoxy-4,4-dimethyl-1-thioxo-1,4-dihydro-5H-[1,2]dithiolo[3,4-c]quinolin-5-yl)-2-oxoethyl]pyrrolidine-2,5-dione 2j,

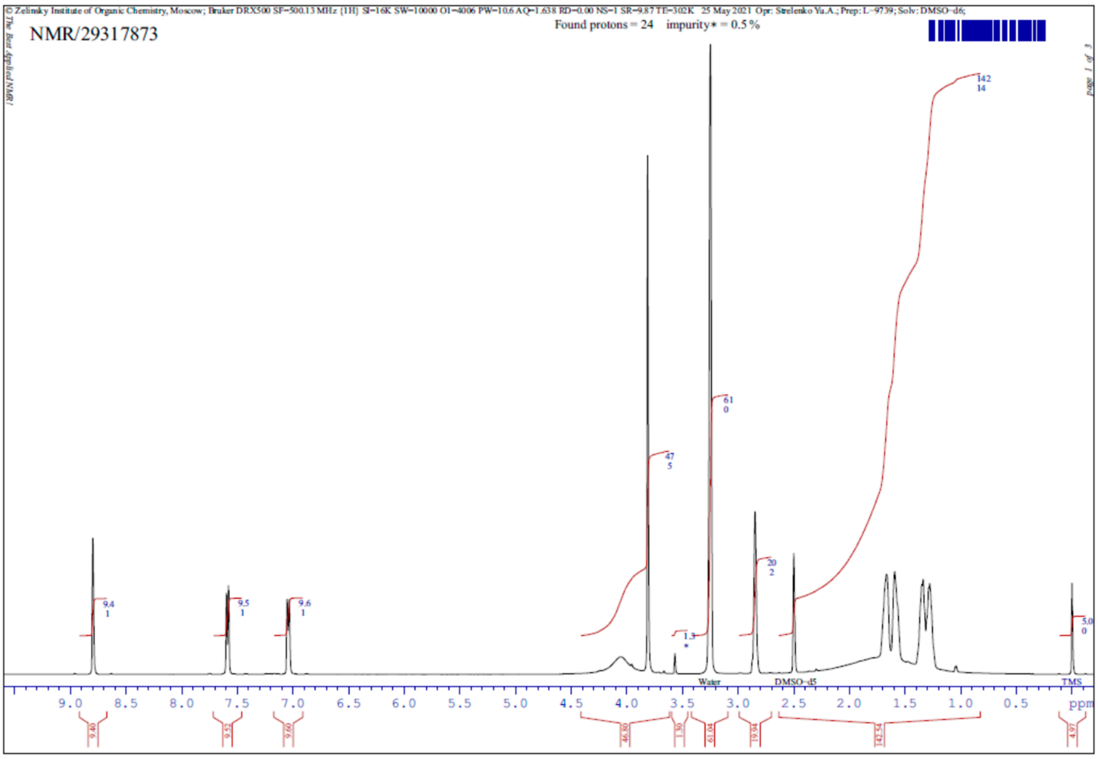

Figure S14: NMR 1H spectra of 2-[2-(8-methoxy-4,4-dimethyl-1-thioxo-1,4-dihydro-5H-[1,2]dithiolo[3,4-c]quinolin-5-yl)-2-oxoethyl]hexahydro-1H-isoindole-1,3(2H)-dione 2k,

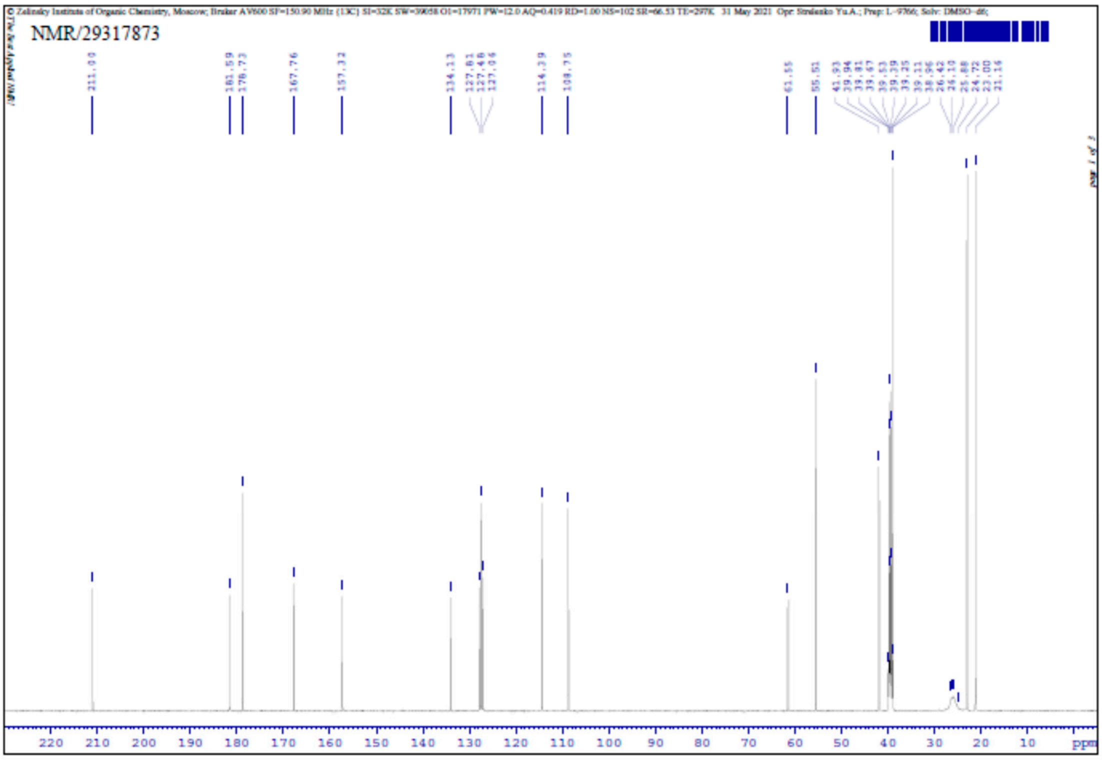

Figure S15: NMR 13C spectra of 2-[2-(8-methoxy-4,4-dimethyl-1-thioxo-1,4-dihydro-5H-[1,2]dithiolo[3,4-c]quinolin-5-yl)-2-oxoethyl]hexahydro-1H-isoindole-1,3(2H)-dione 2k,

**Data Filename**	0035.d	**Sample Name**	
**Sample Type**	Sample	**Position**	Vial 61
**Instrument Name**	Instrument 1	**User Name**	
**Acq Method**	ACN-H2O_60-40.m	**Acquired Time**	3/14/2022 12:37:01 PM
**IRM Calibration Status**	Success	**DA Method**	111.m
**Comment**			

**Sample Group**		**Info.**	
**Stream Name**	LC 1	**Acquisition SW Version**	6200 series TOF/6500 series Q-TOF B.06.01 (B6172 SP1)

User Chromatograms

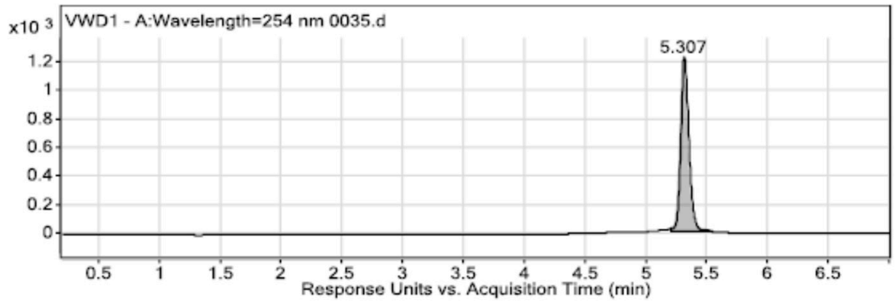

Integration Peak List

Peak	Start	RT	End	Height	Area	Area %
1	5.207	5.307	5.55	1227.8	5933.67	100

User Spectra

Fragmentor Voltage	Collision Energy	Ionization Mode
191	0	ESI

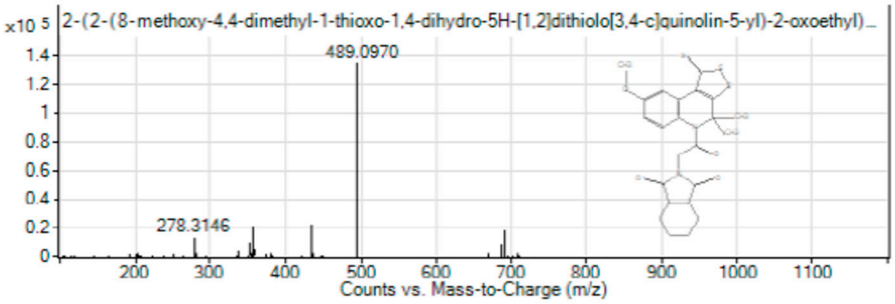

Figure S16: HPLC–HRMS–ESI spectra of 2-[2-(8-methoxy-4,4-dimethyl-1-thioxo-1,4-dihydro-5H-[1,2]dithiolo[3,4-c]quinolin-5-yl)-2-oxoethyl]hexahydro-1H-isoindole-1,3(2H)-dione 2k,

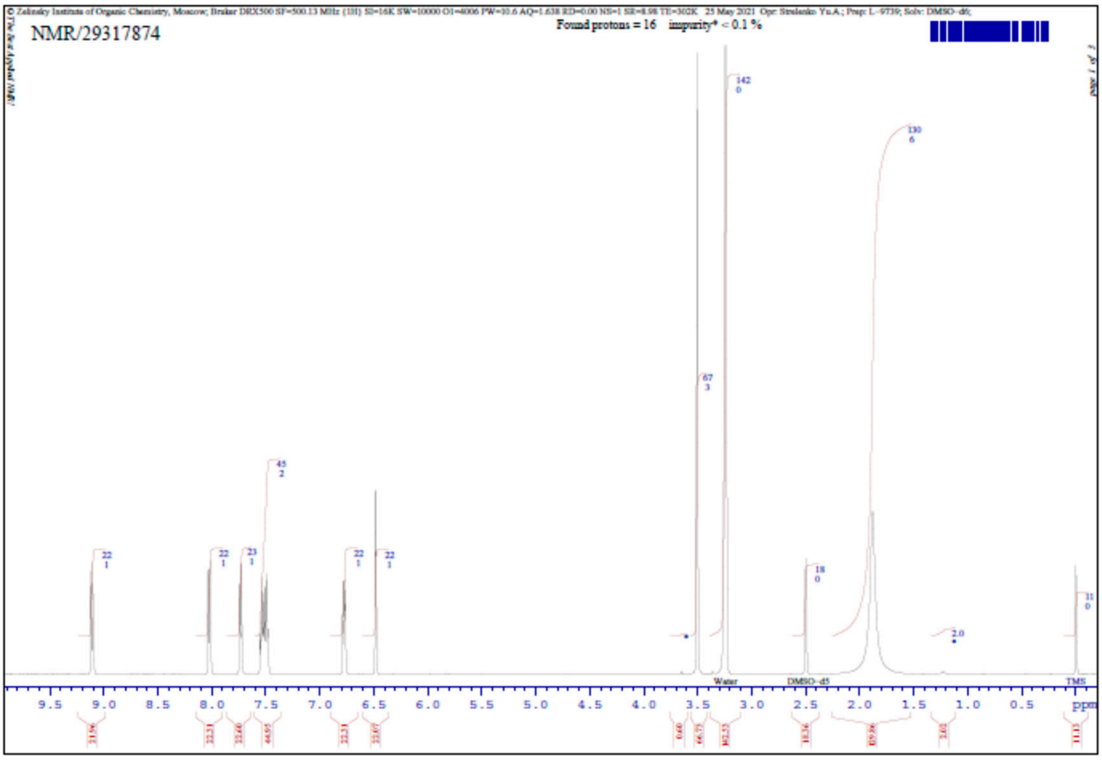

Figure S17: NMR 1H spectra of 5-[(3-chloro-1-benzothien-2-yl)carbonyl]-7-methoxy-4,4-dimethyl-4,5-dihydro-1H-[1,2]dithiolo[3,4-c]quinoline-1-thione 2l,

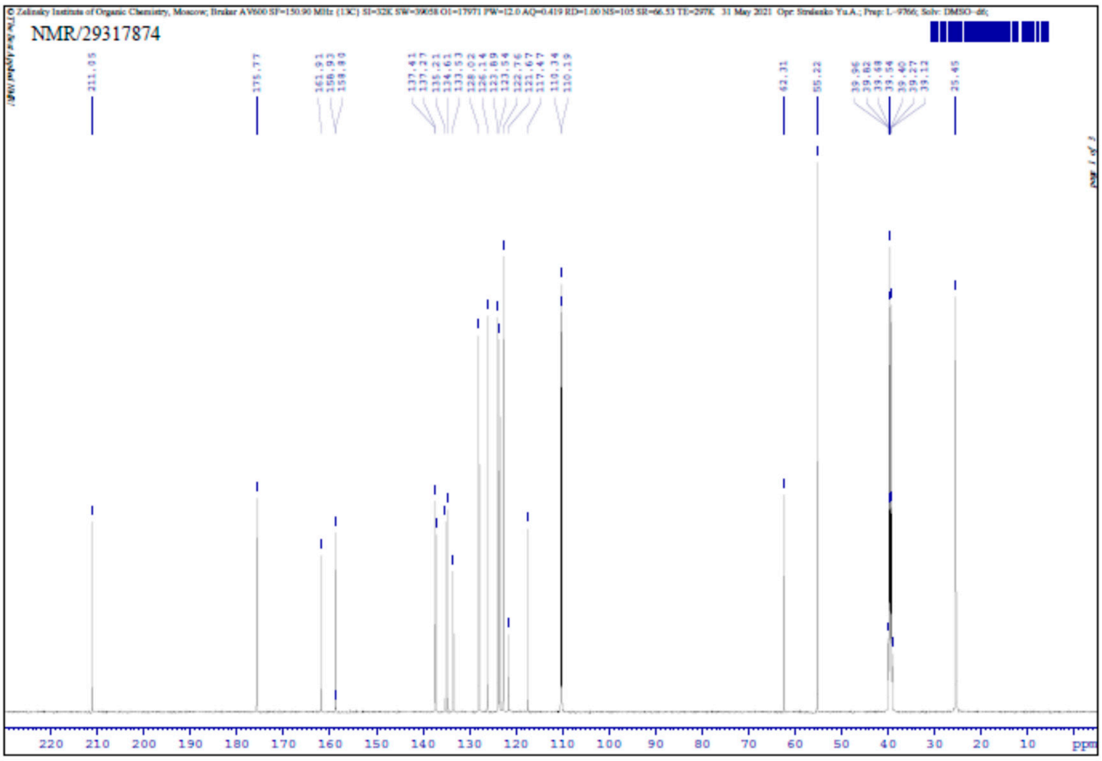

Figure S18: NMR 13C spectra of 5-[(3-chloro-1-benzothien-2-yl)carbonyl]-7-methoxy-4,4-dimethyl-4,5-dihydro-1H-[1,2]dithiolo[3,4-c]quinoline-1-thione 2l,

**Data Filename**	0031.d	**Sample Name**	
**Sample Type**	Sample	**Position**	Vial 57
**Instrument Name**	Instrument 1	**User Name**	
**Acq Method**	ACN-H2O_60-40.m	**Acquired Time**	3/14/2022 11:49:05 AM
**IRM Calibration Status**	Success	**DA Method**	111.m
**Comment**			

**Sample Group**		**Info.**	
**Stream Name**	LC 1	**Acquisition SW Version**	6200 series TOF/6500 series Q-TOF B.06.01 (B6172 SP1)

User Chromatograms

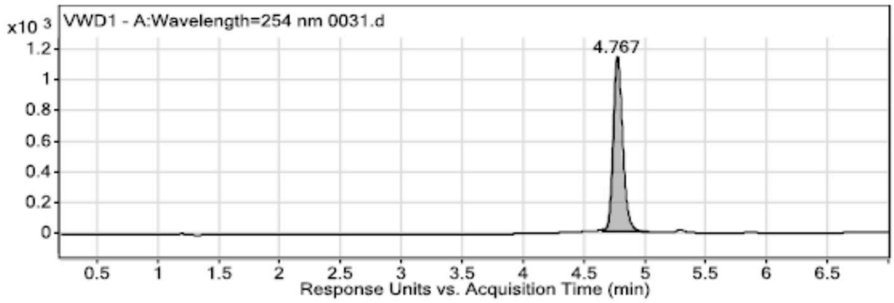

Integration Peak List

Peak	Start	RT	End	Height	Area	Area %
1	4.653	4.767	5.03	1143.6	6139.81	100

User Spectra

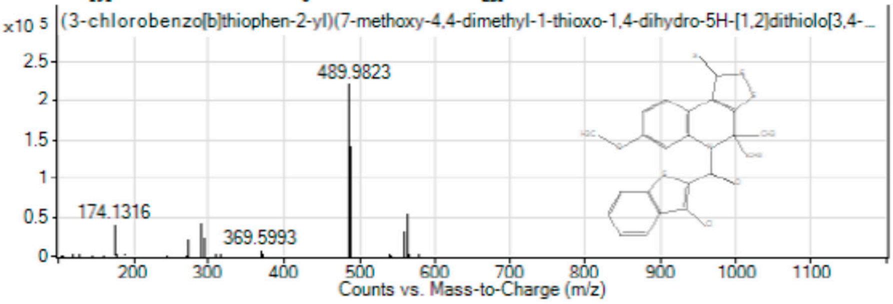

Figure S19: HPLC–HRMS–ESI spectra of 5-[(3-chloro-1-benzothien-2-yl)carbonyl]-7-methoxy-4,4-dimethyl-4,5-dihydro-1H-[1,2]dithiolo[3,4-c]quinoline-1-thione 2l,

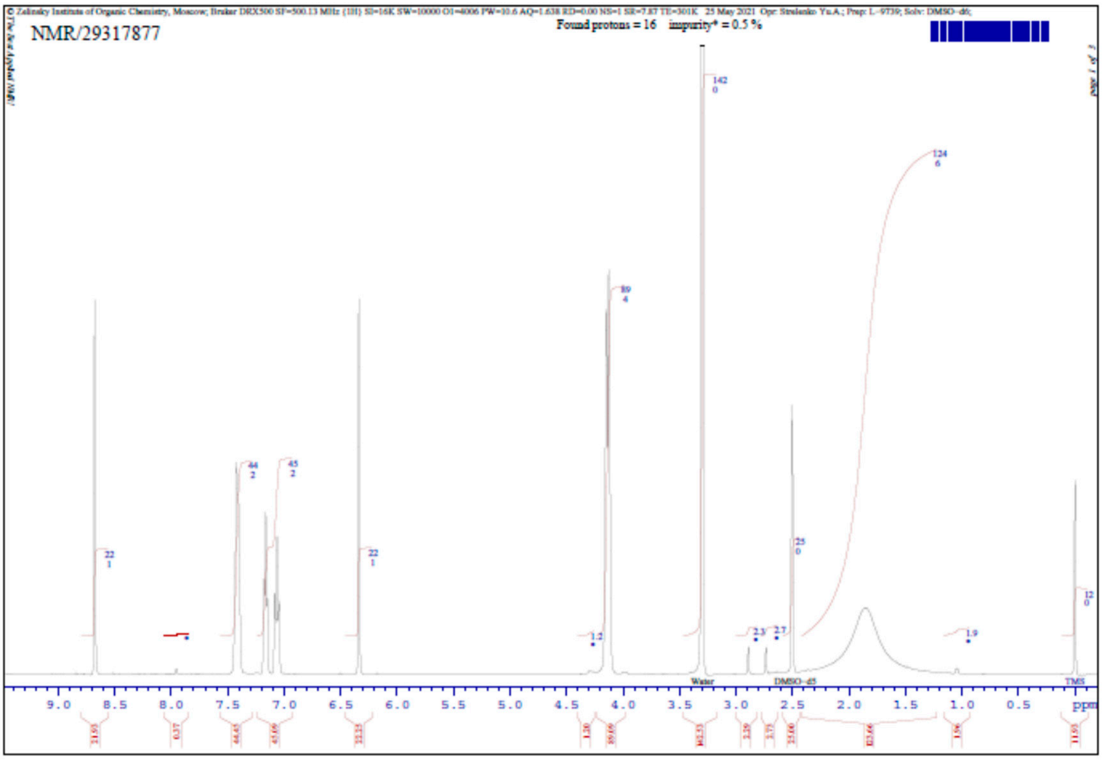

Figure S20: NMR 1H spectra of 5-(2-fluorobenzoyl)-4,4-dimethyl-4,5,8,9-tetrahydro-1H-[1,4]dioxino[2,3-g][1,2]dithiolo[3,4-c]quinoline-1-thione 2m,

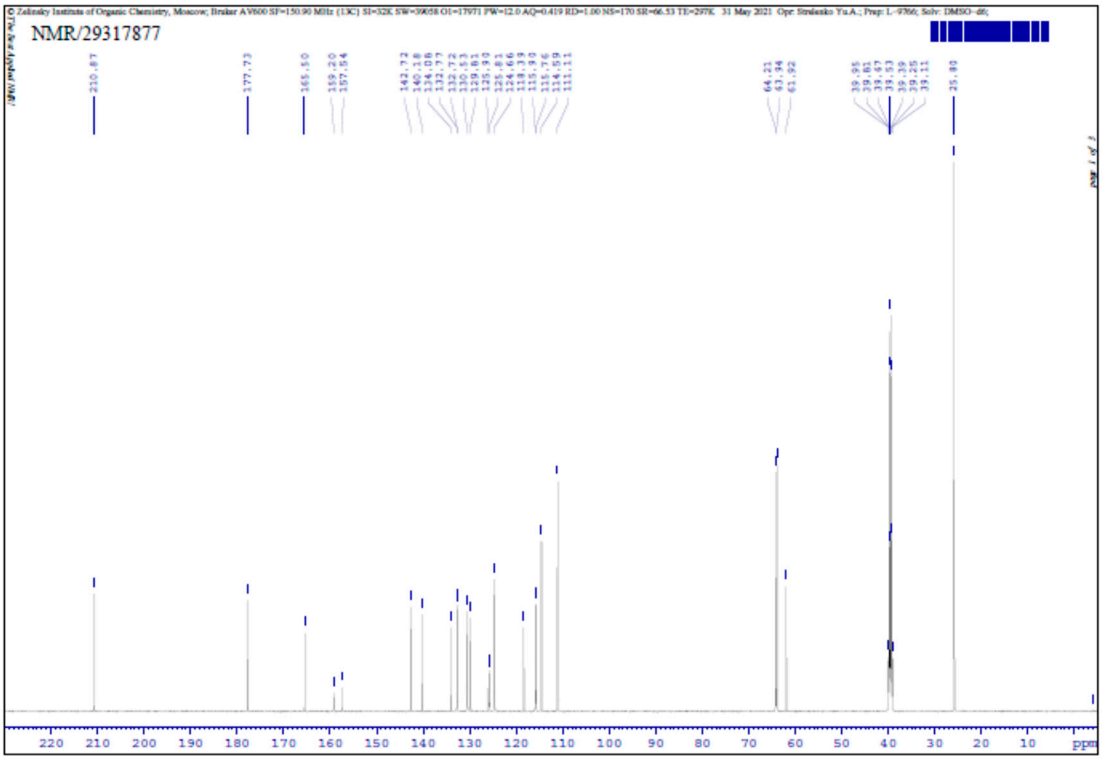

Figure S21: NMR 13C spectra of 5-(2-fluorobenzoyl)-4,4-dimethyl-4,5,8,9-tetrahydro-1H-[1,4]dioxino[2,3-g][1,2]dithiolo[3,4-c]quinoline-1-thione 2m,

**Data Filename**	0001.d	**Sample Name**	
**Sample Type**	Sample	**Position**	Vial 21
**Instrument Name**	Instrument 1	**User Name**	
**Acq Method**	ACN-H2O_60-40.m	**Acquired Time**	3/12/2022 12:05:47 PM
**IRM Calibration Status**	Success	**DA Method**	111.m
**Comment**			

**Sample Group**		**Info.**	
**Stream Name**	LC 1	**Acquisition SW Version**	6200 series TOF/6500 series Q-TOF B.06.01 (B6172 SP1)

User Chromatograms

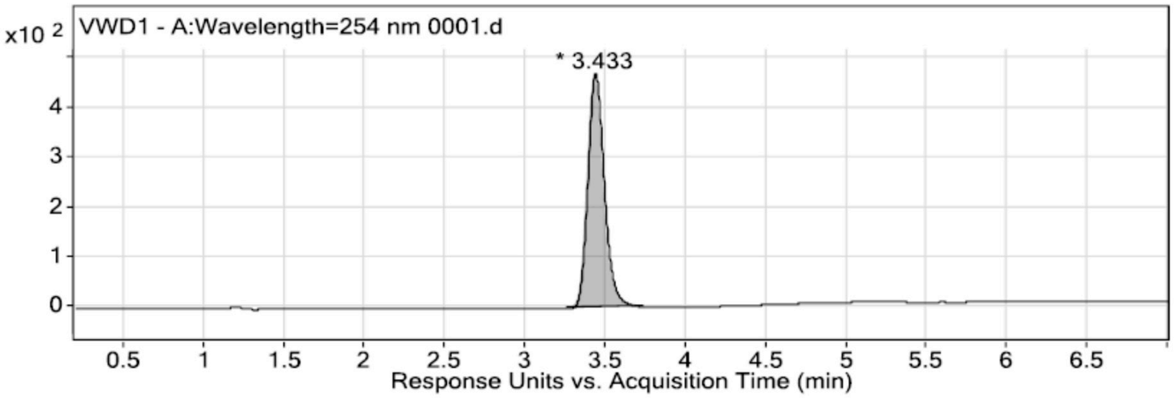

Integration Peak List

Peak	Start	RT	End	Height	Area	Area %
1	3.26	3.433	3.74	469.81	3341.9	100

User Spectra

Fragmentor Voltage	Collision Energy	Ionization Mode
191	0	ESI

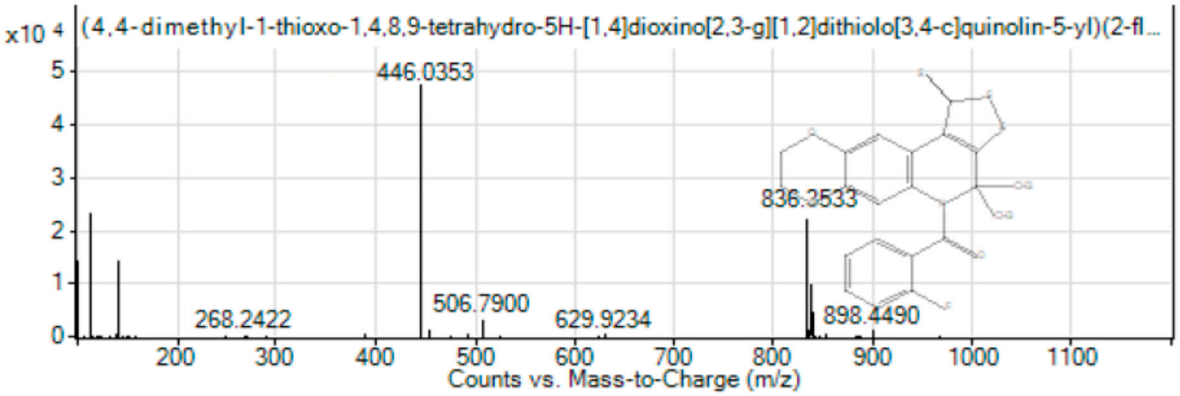

Figure S22: HPLC–HRMS–ESI spectra of 5-(2-fluorobenzoyl)-4,4-dimethyl-4,5,8,9-tetrahydro-1H-[1,4]dioxino[2,3-g][1,2]dithiolo[3,4-c]quinoline-1-thione 2m,

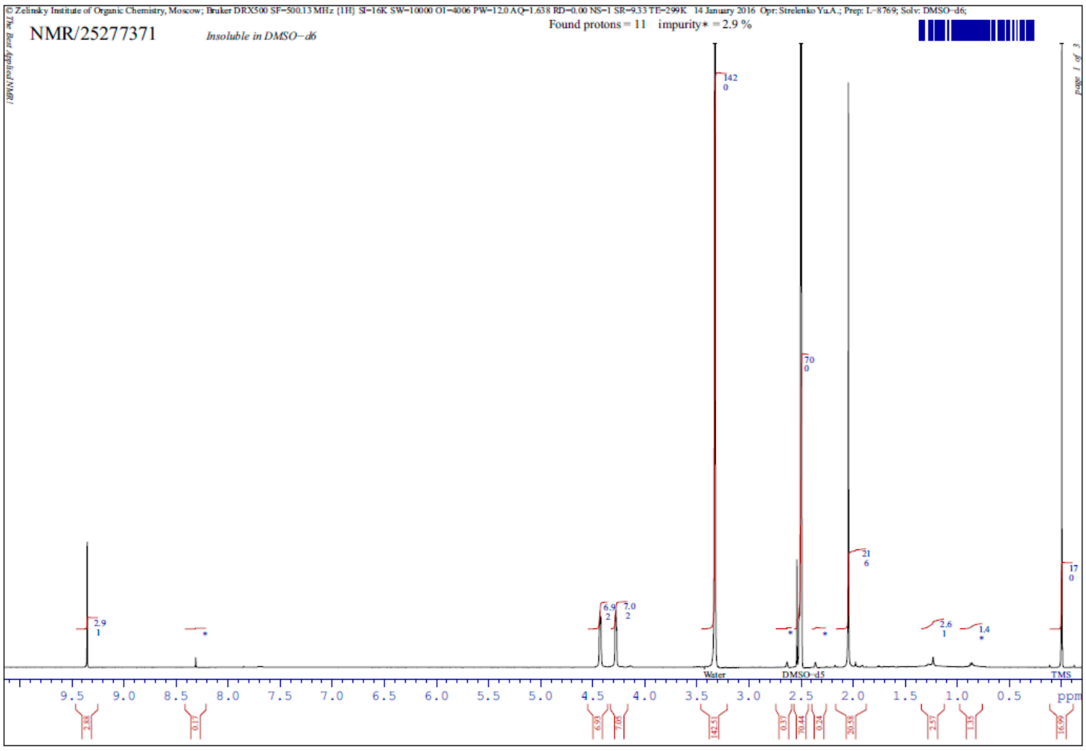

Figure S23: NMR 1H spectra of 8,8-dimethyl-11-thioxo-2,3,8,11-tetrahydro[1,4]dioxino[2,3-g][1,2]dithiolo[3,4-c]pyrrolo[3,2,1-ij]quinoline-5,6-dione 2n,

**Data Filename**	0050.d	**Sample Name**	
**Sample Type**	Sample	**Position**	Vial 76
**Instrument Name**	Instrument 1	**User Name**	
**Acq Method**	ACN-H2O_60-40.m	**Acquired Time**	3/14/2022 3:36:27 PM
**IRM Calibration Status**	Success	**DA Method**	111.m
**Comment**			

**Sample Group**		**Info.**	
**Stream Name**	LC 1	**Acquisition SW Version**	6200 series TOF/6500 series Q-TOF B.06.01 (B6172 SP1)

User Chromatograms

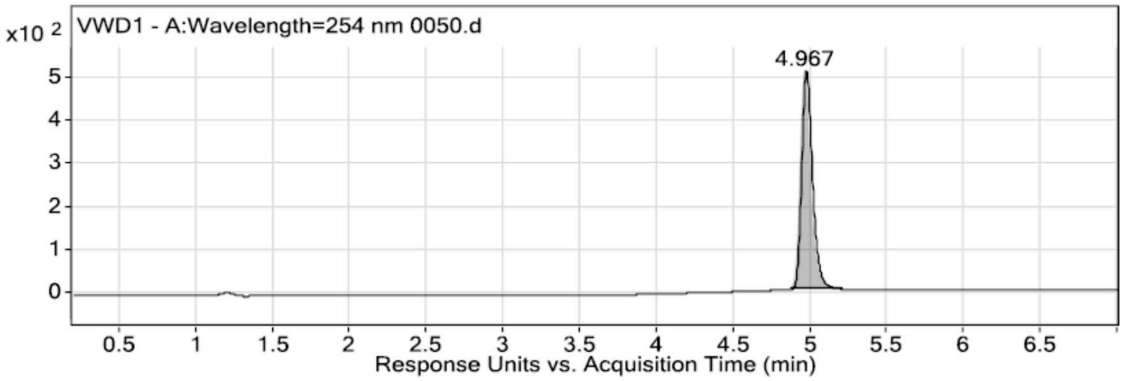

Integration Peak List

Peak	Start	RT	End	Height	Area	Area %
1	4.877	4.967	5.21	505.23	2480.1	100

User Spectra

Fragmentor Voltage	Collision Energy	Ionization Mode
191	0	ESI

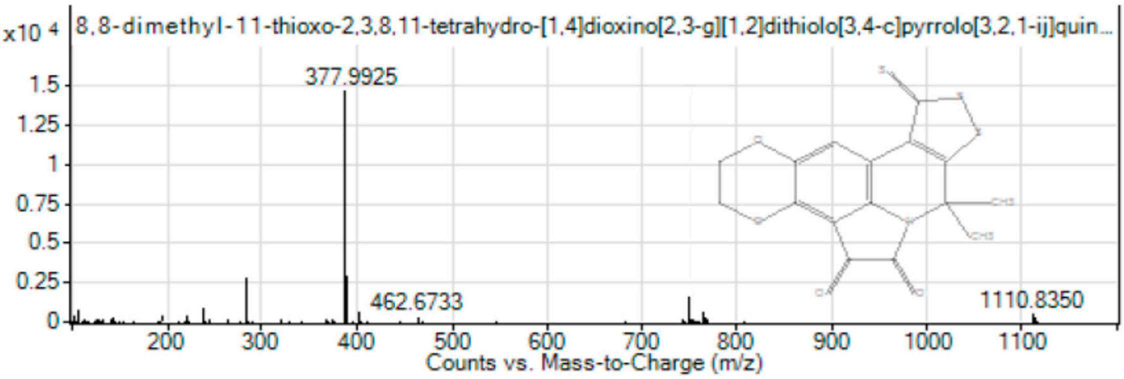

Figure S24: HPLC–HRMS–ESI spectra of 8,8-dimethyl-11-thioxo-2,3,8,11-tetrahydro[1,4]dioxino[2,3-g][1,2]dithiolo[3,4-c]pyrrolo[3,2,1-ij]quinoline-5,6-dione 2n,

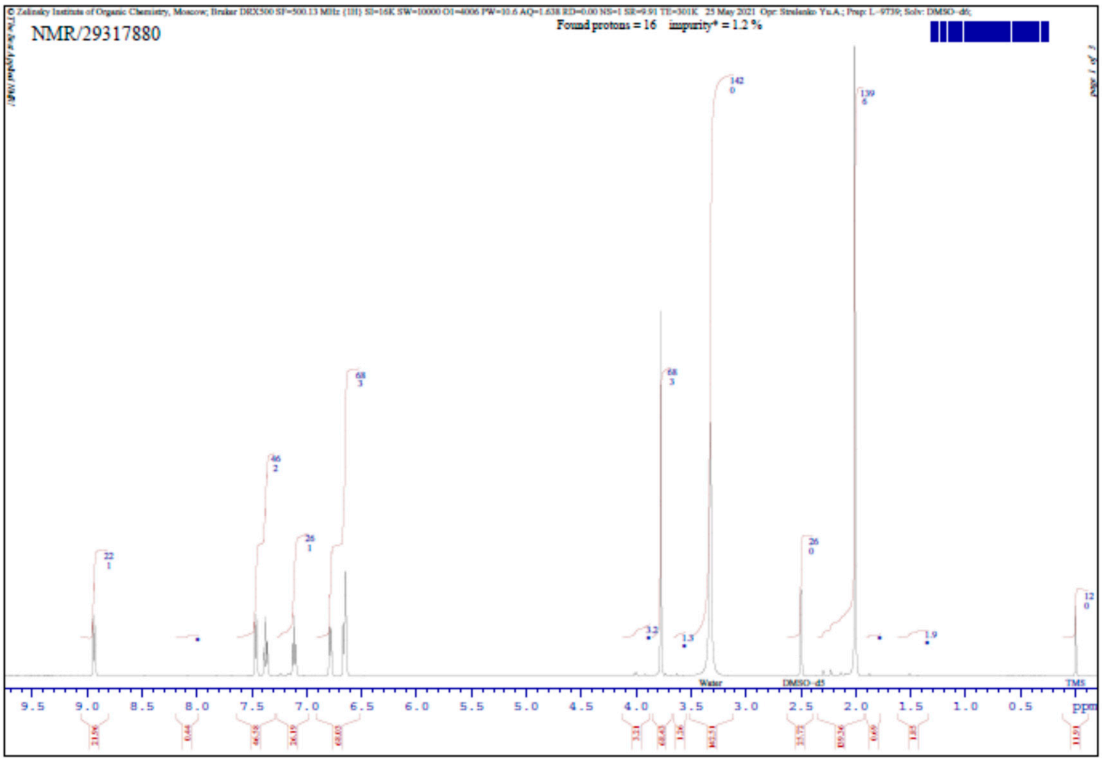

Figure S25: NMR 1H spectra of 10-[(3-methoxyphenyl)imino]-7,7-dimethyl-7,10-dihydro[1,2]dithiolo[3,4-c]pyrrolo[3,2,1-ij]quinoline-4,5-dione 2q,

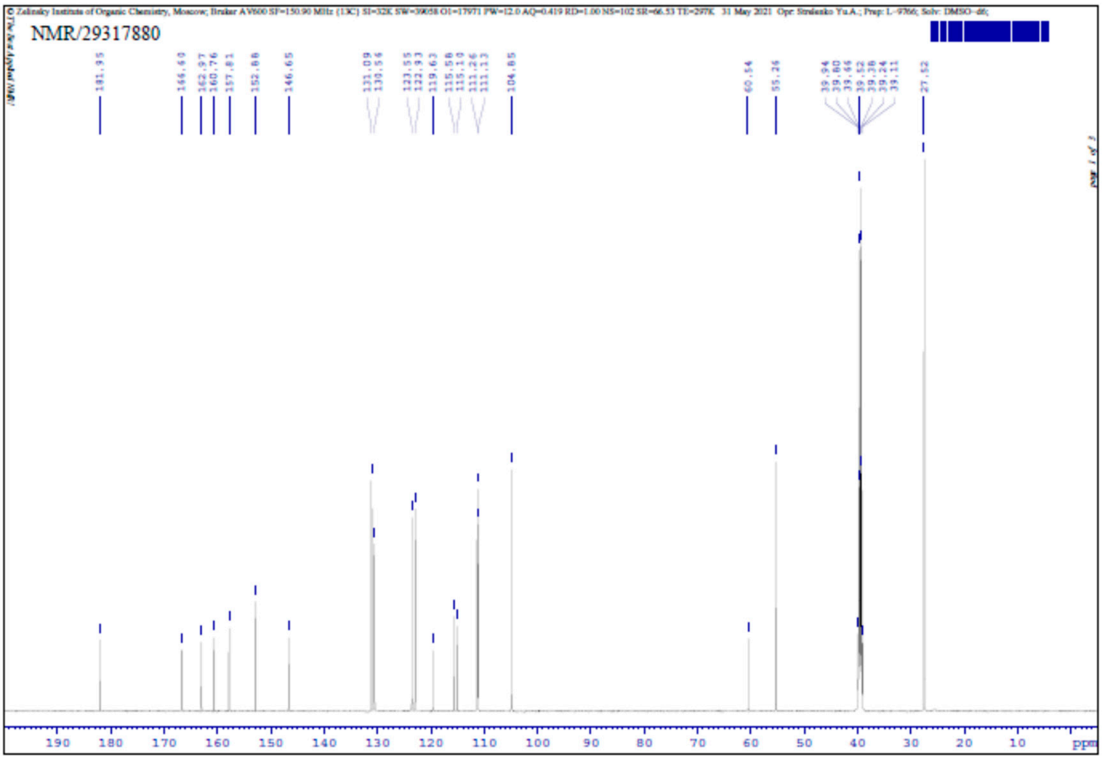

Figure S26: NMR 13C spectra of 10-[(3-methoxyphenyl)imino]-7,7-dimethyl-7,10-dihydro[1,2]dithiolo[3,4-c]pyrrolo[3,2,1-ij]quinoline-4,5-dione 2q,

**Data Filename**	0024.d	**Sample Name**	
**Sample Type**	Sample	**Position**	Vial 44
**Instrument Name**	Instrument 1	**User Name**	
**Acq Method**	ACN-H2O_60-40.m	**Acquired Time**	3/12/2022 4:44:26 PM
**IRM Calibration Status**	Success	**DA Method**	111.m
**Comment**			

**Sample Group**		**Info.**	
**Stream Name**	LC 1	**Acquisition SW Version**	6200 series TOF/6500 series Q-TOF B.06.01 (B6172 SP1)

User Chromatograms

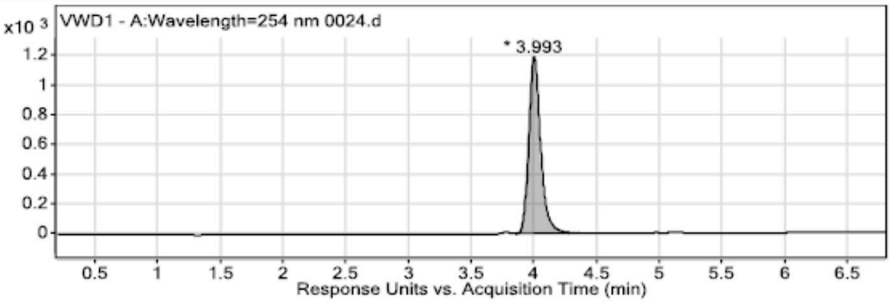

Integration Peak List

Peak	Start	RT	End	Height	Area	Area %
1	3.86	3.993	4.363	1198.81	8022.31	100

User Spectra

Fragmentor Voltage	Collision Energy	Ionization Mode
191	0	ESI

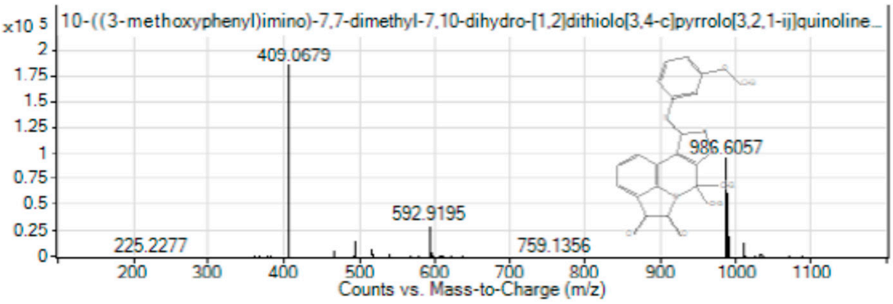

Figure S27: HPLC–HRMS–ESI spectra of 10-[(3-methoxyphenyl)imino]-7,7-dimethyl-7,10-dihydro[1,2]dithiolo[3,4-c]pyrrolo[3,2,1-ij]quinoline-4,5-dione 2q.
